# 

**Published:** 1988-05

**Authors:** 


					Editor
Mr M. G. Wilson
40 Coombe Lane, Bristol BS9 2BJ
Tel: 0272-682320
Assistant Editor
Dr P. R. Goddard
Editorial Committee
Mr B. P. Jones (Secretary),
Medical Librarian, Bristol University
Dr J. L. Burton
Correspondents
Dr A. E. Adam (Taunton)
Mr D. C. C. Bartolo
Mr M. L. Crosfill (Penzance)
Dr J. D. Davies
Dr J. Jancar
Professor D. J. Pereira Gray (Exeter)
Dr H. G. Mather
Professor G. M. Stirratt
Mr C. Teasdale (Plymouth)
Dr J. L. G. Thomson
Dr M. J. Whitfield
Professor G. K. Wilcock
Professor R. C. N. Williamson
BRISTOL MEDICO-CHIRURGICAL SOCIETY
President
Dr H. M. Norman
Secretary
Mr R. N. Baird,
23 Old Sneed Park, Bristol BS9 1RG
Treasurer
Dr N. C. Tricks,
The Mill, Wrington, Bristol
Published by
Clinical Press,
Greinton House,
8 Exeter Buildings,
Redland,
Bristol BS6 6TH
Typesetting by
Avon Communications Ltd.,
Avon House, Nail sea, Bristol BS19 2D J
Printed by
Suttons, Paignton
?Establ ished 1883i
BRISTOL
MEDICO
CHIRl IRGICAT.
K3URNAL
MAY 1988
Volume 103 (ii)
Published quarterly and distributed free to all doctors in
the West of England. Overseas subscription ?12.
NOTICE TO CONTRIBUTORS
Contributions are invited especially from West of England authors on a variety of subjects, e.g. Original articles relating to the practice
of medicine, reports on the contemporary medical scene, obituaries, historical accounts, recollections of colleagues and events worthy
to be remembered and liable to be forgotten. An article of 2,000 words with 2 illustrations will occupy 2 pages and this is a suggested,
though by no means an obligatory length. Articles are preferred in the form proposed by the International Committee of Medical
Editors (Vancouver System)?pamphlet obtainable from Editor of BMJ., BMA. House, Tavistock Square, London, WC1H 9JR.
J) ^  v^s
r/ ^ ^
ntca/
An independent new force in medical publishing Medical authors and editors in the West
and Wales! Clinical Press has been established especially to serve you.
If you are thinking of writing a book, producing conference proceedings, editing a
journal, or simply want advice on publishing we would be glad to hear from you.
We already have a growing list of eminent authors. Why not join them?
Clinical Press combines proven local editorial and publishing skills with
international marketing connections.
\ f
Greinton House, 8, Exeter Buildings, Redland, Bristol. BS6 6TH
Telephone: Bristol (0272) 854885 or 237709
24

				

